# PD61 Economic And Health Burden Characterization of Migraine Population At The FUSAT Hospital: An Opportunity For Innovative Tailored Intervention

**DOI:** 10.1017/S0266462325101931

**Published:** 2025-12-29

**Authors:** Ruben Rojas, Luis Arriagada, Gabriel Pinto, Carolina Cruz, Patricia Fernandez, Vicente Garzaro, Tomás Abbott, Monica Velasquez

## Abstract

**Introduction:**

Migraine is a neurological condition with significant impacts on quality of life, productivity, and healthcare costs. In Chile, understanding the economic and health burden of migraine in a closed health center, such as the FUSAT hospital, is essential to generate evidence of the burden associated with the pathology and to define effective strategies for its management.

**Methods:**

This retrospective study used clinical and claims data collected at the FUSAT Hospital between 2018 and 2023. Patients with diagnosis of migraine were identified and information from outpatient consultations, emergency care, hospitalizations, and sick leave were analyzed. Costs were adjusted to 2023 values and comorbidities were evaluated

**Results:**

Migraine diagnosis was found in 2.8 percent (n=395) of the FUSAT population, predominantly in women aged 45 to75 years. Hypertension (30.1%), anxiety (28.6%), and depression (12%) were frequent among migraine patients. Regarding healthcare costs, the annual expenditure for a patient with migraine was 25 percent higher than the costs reported for patients without migraine. Concerning sick leaves, 12 percent of the migraine population reported at least one sick leave, with an expected cost per patient of CLP2,854,700 (USD2,854), which represents a 20 percent increase in comparison to the average FUSAT patient.

**Conclusions:**

Migraine represents a significant economic and social burden in the FUSAT, mainly affecting women between the ages of 45 and 74 years. The study highlighted the need for specific, tailored interventions focused on optimizing resource usage and health outcomes. Furthermore, a risk-sharing model for rimegepant, based on performance, was proposed for the preventive management of migraine in FUSAT.

